# Clear cell meningiomas are defined by a highly distinct DNA methylation profile and mutations in *SMARCE1*

**DOI:** 10.1007/s00401-020-02247-2

**Published:** 2020-12-14

**Authors:** Philipp Sievers, Martin Sill, Christina Blume, Arnault Tauziede-Espariat, Daniel Schrimpf, Damian Stichel, David E. Reuss, Helin Dogan, Christian Hartmann, Christian Mawrin, Martin Hasselblatt, Walter Stummer, Uta Schick, Jürgen Hench, Stephan Frank, Ralf Ketter, Leonille Schweizer, Jens Schittenhelm, Stéphanie Puget, Sebastian Brandner, Zane Jaunmuktane, Benno Küsters, Zied Abdullaev, Melike Pekmezci, Matija Snuderl, Miriam Ratliff, Christel Herold-Mende, Andreas Unterberg, Kenneth Aldape, David W. Ellison, Pieter Wesseling, Guido Reifenberger, Wolfgang Wick, Arie Perry, Pascale Varlet, Stefan M. Pfister, David T. W. Jones, Andreas von Deimling, Felix Sahm, The German Consortium “Aggressive Meningiomas”

**Affiliations:** 1 Department of Neuropathology, Institute of Pathology, University Hospital Heidelberg, Heidelberg, Germany; 2 Clinical Cooperation Unit Neuropathology, German Consortium for Translational Cancer Research (DKTK), German Cancer Research Center (DKFZ), Heidelberg, Germany; 3 Hopp Children’s Cancer Center Heidelberg (KiTZ), Heidelberg, Germany; 4 Division of Pediatric Neurooncology, German Cancer Consortium (DKTK), German Cancer Research Center (DKFZ), Heidelberg, Germany; 5 Bioinformatics and Omics Data Analytics, German Cancer Research Center (DKFZ), Heidelberg, Germany; 6 Department of Neuropathology, GHU Paris Psychiatry and Neurosciences, Sainte-Anne Hospital, Paris, France; 7 Department of Neuropathology, Institute of Pathology, Hannover Medical School (MHH), Hannover, Germany; 8 Department of Neuropathology, Otto-Von-Guericke University, Magdeburg, Germany; 9 Institute of Neuropathology, University Hospital Münster, Münster, Germany; 10 Department of Neurosurgery, University Hospital Münster, Münster, Germany; 11 Department of Neurosurgery, Clemenshospital Münster, Münster, Germany; 12 Institute for Medical Genetics and Pathology, University Hospital Basel, Basel, Switzerland; 13 Department of Neurosurgery, University Hospital Homburg Saar, Homburg, Germany; 14 Department of Neuropathology, Charité-Universitätsmedizin Berlin, Corporate Member of Freie Universität Berlin, Humboldt-Universität zu Berlin, and Berlin Institute of Health, Berlin, Germany; 15 German Cancer Consortium (DKTK), Partner Site Berlin, German Cancer Research Center (DKFZ), Heidelberg, Germany; 16 Department of Neuropathology, University of Tübingen, Tübingen, Germany; 17 Department of Pediatric Neurosurgery, Hôpital Necker-Enfants Malades, APHP, Université de Paris, Paris, France; 18 Division of Neuropathology, National Hospital for Neurology and Neurosurgery, University College London Hospitals NHS Foundation Trust, Queen Square, London, UK; 19 Department of Neurodegenerative Disease, UCL Queen Square Institute of Neurology, Queen Square, London, UK; 20 Department of Clinical and Movement Neurosciences and Queen Square Brain Bank for Neurological Disorders, Queen Square Institute of Neurology, University College London, London, UK; 21 Department of Pathology, Radboud University Medical Center, Nijmegen, the Netherlands; 22 Laboratory of Pathology, Center for Cancer Research, National Cancer Institute, National Institutes of Health, Bethesda, MD USA; 23 Department of Pathology, University of California, San Francisco, CA USA; 24 Department of Pathology, NYU Langone Medical Center, New York, NY USA; 25 Department of Neurosurgery, University Medical Centre Mannheim, University of Heidelberg, Mannheim, Germany; 26 Division of Experimental Neurosurgery, Department of Neurosurgery, University Hospital Heidelberg, Heidelberg, Germany; 27 Department of Neurosurgery, University Hospital Heidelberg, Heidelberg, Germany; 28 Department of Pathology, St. Jude Children’s Research Hospital, Memphis, TN USA; 29 Department of Pathology, Amsterdam University Medical Centers, Location VUmc and Brain Tumor Center Amsterdam, Amsterdam, The Netherlands; 30 Princess Máxima Center for Pediatric Oncology, Utrecht, The Netherlands; 31 Institute of Neuropathology, Heinrich Heine University, Düsseldorf, Germany; 32 German Cancer Consortium (DKTK), Partner Site Essen/Düsseldorf, Germany; 33 Clinical Cooperation Unit Neurooncology, German Consortium for Translational Cancer Research (DKTK), German Cancer Research Center (DKFZ), Heidelberg, Germany; 34 Department of Neurology and Neurooncology Program, National Center for Tumor Diseases, Heidelberg University Hospital, Heidelberg, Germany; 35 Department of Neurological Surgery, University of California, San Francisco, CA USA; 36 Department of Pediatric Oncology, Hematology, Immunology and Pulmonology, University Hospital Heidelberg, Heidelberg, Germany; 37 Pediatric Glioma Research Group, German Cancer Research Center (DKFZ), Heidelberg, Germany

**Keywords:** Brain tumor, Meningioma, Clear cell, *SMARCE1*, DNA methylation profile

## Abstract

Clear cell meningioma represents an uncommon variant of meningioma that typically affects children and young adults. Although an enrichment of loss-of-function mutations in the *SMARCE1* gene has been reported for this subtype, comprehensive molecular investigations are lacking. Here we describe a molecularly distinct subset of tumors (*n* = 31), initially identified through genome-wide DNA methylation screening among a cohort of 3093 meningiomas, of which most were diagnosed histologically as clear cell meningioma. This cohort was further supplemented by an additional 11 histologically diagnosed clear cell meningiomas for analysis (*n* = 42). Targeted DNA sequencing revealed *SMARCE1* mutations in 33/34 analyzed samples, accompanied by a nuclear loss of expression determined via immunohistochemistry and a decreased SMARCE1 transcript expression in the tumor cells. Analysis of time to progression or recurrence of patients within the clear cell meningioma group (*n* = 14) in comparison to those with meningioma WHO grade 2 (*n* = 220) revealed a similar outcome and support the assignment of WHO grade 2 to these tumors. Our findings indicate the existence of a highly distinct epigenetic signature of clear cell meningiomas, separate from all other variants of meningiomas, with recurrent mutations in the *SMARCE1* gene. This suggests that these tumors may arise from a different precursor cell population than the broad spectrum of the other meningioma subtypes.

**Supplementary Information:**

The online version contains supplementary material available at 10.1007/s00401-020-02247-2.

## Introduction

Meningioma is the most common primary central nervous system (CNS) neoplasm, accounting for about a third of all brain tumors [10, 16]. Clear cell meningioma represents an uncommon variant of meningioma that typically affects children and young adults [12, 21–23, 26]. Histologically, it is characterized by sheets of rounded or polygonal clear cells and perivascular and interstitial collagen [10]. Clear cell meningioma is associated with a more aggressive behavior [29] and is, therefore, classified as a World Health Organization (WHO) grade 2 tumor [10]. Genetically, an enrichment of loss-of-function mutations in the *SMARCE1* gene encoding a subunit of the SWI/SNF chromatin remodeling complex has been reported for this meningioma subtype [22, 23]. *NF2* mutations, common in other meningiomas, are rare in this subtype [21]. Yet, it remains elusive whether clear cell meningioma is merely a morphological variant (albeit with prognostic implications) that can in principle be associated with various driving alterations, or whether the predominance of *SMARCE1* alterations rather points to a defining set of molecular underpinnings unique to these tumors.

Investigating the DNA methylation landscape of meningiomas, we identified a cluster of 31 tumor samples in a cohort of 3093 meningiomas (~ 1%) that formed a highly distinct group, well detached from the other meningiomas, of which most cases were diagnosed histologically as clear cell meningioma. Based on this observation we collected additional morphologically identified clear cell meningiomas (*n* = 11) and performed further molecular workup on the whole cohort (*n* = 42) using DNA methylation profiling, targeted next-generation DNA sequencing and RNA sequencing.

## Materials and methods

### Sample collection

Tumor samples and retrospective clinical data from 42 patients were provided by multiple national and international collaborating centers and collected at the Department of Neuropathology of the University Hospital Heidelberg (Germany). Case selection was based on unsupervised hierarchical clustering of genome-wide DNA methylation data in a cohort of 3,093 meningiomas that revealed a molecularly distinct group of tumors comprising 31 samples, of which most were diagnosed histologically as clear cell meningioma. Additionally, 11 histologically diagnosed clear cell meningiomas were subsequently integrated into analyses to verify that the specific cluster is made up by this subtype of meningioma. Analysis of tissue and clinical data was performed in accordance with local ethics regulations. Clinical details of the patients are listed in Supplementary Table 1 (online resource).

### Immunohistochemistry

Immunohistochemistry was performed on a Ventana BenchMark ULTRA Immunostainer (Ventana Medical Systems, Tucson, AZ, USA) for all cases with sufficient tissue (for EMA and SSTR2A *n* = 22; for SMARCE1 *n* = 25; for Ki-67 *n* = 26). Antibodies were directed against: epithelial membrane antigen (EMA; Clone GP1.4, mouse monoclonal, dilution 1:1000, Thermo Fisher Scientific, Fremont, CA, USA), somatostatin receptor 2A (SSTR2A; SS-8000-RM, rabbit monoclonal, dilution 1:10, Biotrend, Cologne, Germany), Ki-67 (clone MIB-1, mouse monoclonal, 1:100 dilution, Dako Agilent, Santa Clara, CA, USA) and SMARCE1 (HPA003916, rabbit polyclonal, dilution 1:700, Sigma-Aldrich, St. Louis, MO, USA).

### DNA methylation array processing and copy number profiling

Genomic DNA was extracted from formalin-fixed and paraffin-embedded (FFPE) tissue samples. DNA methylation profiling of all samples was performed using the Infinium MethylationEPIC (850k) BeadChip (Illumina, San Diego, CA, USA) or Infinium HumanMethylation450 (450k) BeadChip array (Illumina) as previously described [2]. All computational analyses were performed in R version 3.3.1 (R Development Core Team, 2016; https://www.R-project.org). Copy-number variation analysis from 450k and EPIC methylation array data was performed using the conumee Bioconductor package version 1.12.0. Raw signal intensities were obtained from IDAT-files using the minfi Bioconductor package version 1.21.4 [1]. Illumina EPIC samples and 450k samples were merged to a combined data set by selecting the intersection of probes present on both arrays (combineArrays function, minfi). Each sample was individually normalized by performing a background correction (shifting of the 5% percentile of negative control probe intensities to 0) and a dye-bias correction (scaling of the mean of normalization control probe intensities to 10,000) for both color channels. Subsequently, a correction for the array type (450k/EPIC) was performed by fitting univariable, linear models to the log2-transformed intensity values (removeBatchEffect function, limma package version 3.30.11). The methylated and unmethylated signals were corrected individually. Beta-values were calculated from the retransformed intensities using an offset of 100 (as recommended by Illumina). All samples were checked for duplicates by pairwise correlation of the genotyping probes on the 450k/850k array. To perform unsupervised non-linear dimension reduction, the remaining probes after standard filtering [2] were used to calculate the 1-variance weighted Pearson correlation between samples. The resulting distance matrix was used as input for t-SNE analysis (t-distributed stochastic neighbor embedding; Rtsne package version 0.13). The following non-default parameters were applied: theta = 0, pca = F, max_iter = 15,000 perplexity = 20.

### Targeted next-generation DNA sequencing and mutational analysis

Genomic DNA was extracted from FFPE tumor tissue samples of 34 patients within the cohort using the automated Maxwell system with the Maxwell 16 FFPE Plus LEV DNA Purification Kit (Promega, Madison, WI, USA), according to the manufacturer’s instructions. Matched normal DNA was extracted from blood samples of four of the affected individuals using the Maxwell 16 Blood DNA Purification Kit (Promega). Capture-based next-generation DNA sequencing was performed on a NextSeq 500 instrument (Illumina) as previously described [18] using a custom brain tumor panel covering the entire coding and selected intronic and promoter regions of 130 genes of particular relevance in central nervous system tumors. Reads were aligned against the reference genome (GRch37).

### RNA sequencing and analysis

RNA was extracted from FFPE tissue samples using the automated Maxwell system with the Maxwell 16 LEV RNA FFPE Kit (Promega, Madison, WI, USA), according to the manufacturer’s instructions. Transcriptome analysis using messenger RNA (mRNA) sequencing of samples for which RNA of sufficient quality and quantity was available (clear cell meningioma (*n* = 15), *NF2*-mutant meningioma (*n* = 12)) was performed on a NextSeq 500 (Illumina) as previously described [24]. Fastq files from transcriptome sequencing were used for de novo annotation of fusion transcripts using the defuse [14] and arriba (https://github.com/suhrig/arriba/) algorithms with standard parameters. Alignment to the human genome (GRCh37) was performed with the STAR aligner [4] and reads were then quantified using RSEM [9]. All further analysis was performed in R (version 3.6.0; R Core Team, 2019) using the DESeq2 package (version 1.26.0) [11]. Principal Component Analysis (PCA) was performed after variance stabilizing transformation of the count data and normalization with respect to library size. Similarities between samples were determined by computing Manhattan distances on the variance stabilized data followed by unsupervised hierarchical clustering. Differential expression testing was performed on raw count data after fitting a negative binomial model. *P*-values were adjusted for multiplicity by applying the Benjamini–Hochberg correction.

### Statistical analysis

Statistical analysis was performed using GraphPad Prism 8 (GraphPad Software, La Jolla, CA, USA). Data on survival could be retrospectively retrieved for 14 patients. Distribution of time to progression or recurrence (TTP) after surgery was estimated by the Kaplan–Meier method and compared between groups with the log-rank test. *P*-values below 0.05 were considered significant.

## Results

### DNA methylation profiling reveals a highly distinct epigenetic signature of clear cell meningioma

**Fig. 1:**
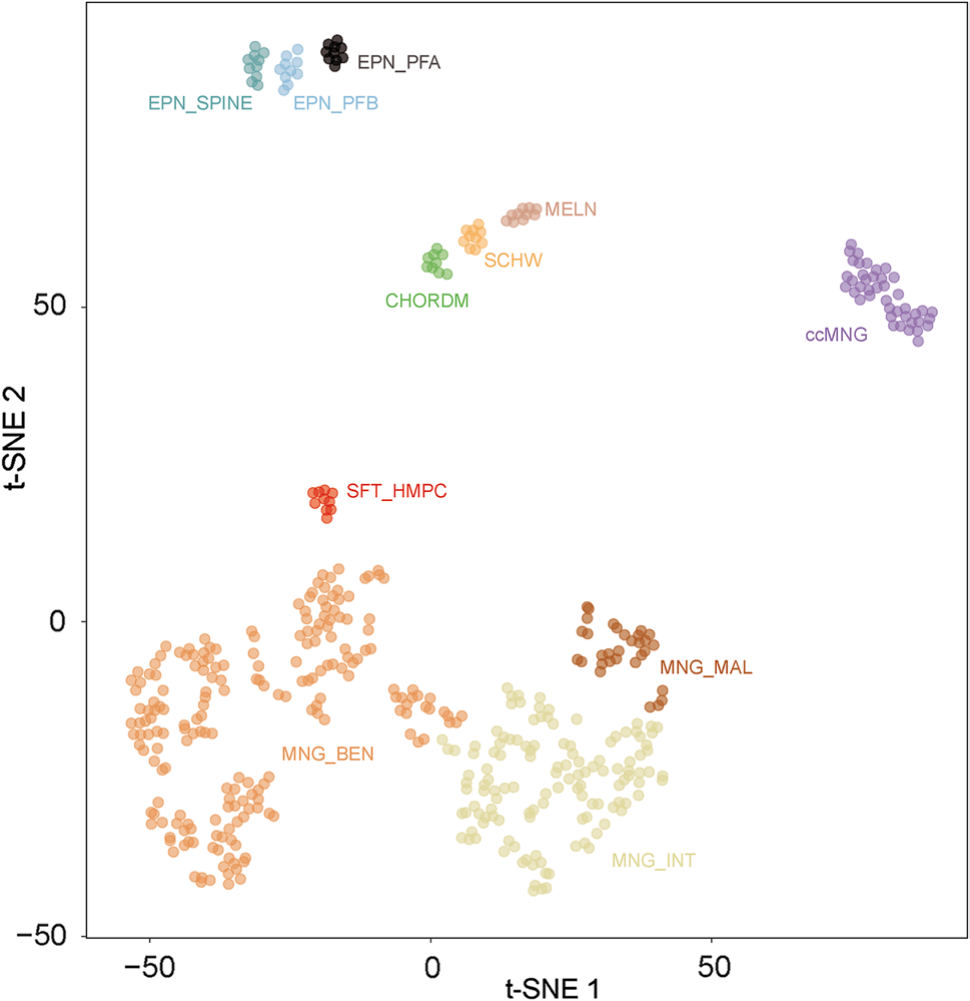
t-distributed stochastic neighbor embedding (t-SNE) analysis of DNA methylation profiles of the 42 clear cell meningiomas (ccMNG) alongside selected reference samples. Reference DNA methylation classes: meningioma benign (MNG_BEN), meningioma intermediate (MNG_INT), meningioma malignant (MNG_MAL), ependymoma posterior fossa group A (EPN_PFA), ependymoma posterior fossa group B (EPN_PFB), ependymoma spinal (EPN_SPINE), solitary fibrous tumor/hemangiopericytoma (SFT_HMPC), schwannoma (SCHW), chordoma (CHORD) and secondary/metastatic melanoma (MELN)

Investigating the DNA methylation landscape of meningiomas, we identified a group of 31 tumor samples in a cohort of 3093 meningiomas that formed a highly distinct cluster, well detached from the other meningiomas (Suppl. Figure 1, online resource). Intriguingly, most (18/31) cases in this initial cluster had been diagnosed histologically as clear cell meningioma, followed by atypical meningioma as the second most common diagnosis (Suppl. Table 1, online resource). Based on this observation we collected additional clear cell meningiomas based on their morphology (*n* = 11) that subsequently also all grouped with this specific cluster by DNA methylation profiling. A more focused t-distributed stochastic neighbor embedding (t-SNE) analysis of DNA methylation patterns confirmed the distinct nature of this group (Fig. 1). In contrast, none of the other meningioma subtypes with characteristic alterations, e.g. mutations in *NF2*, *TRAF7/KLF4* or *BAP1*, or *YAP1* fusion seem to form independent epigenetic clusters (Suppl. Figure 2, online resource). Analysis of copy number profiles derived from DNA methylation data revealed recurrent chromosomal aberrations within the clear cell meningioma group including chromosome 17q (segmental) loss, chromosome 6q loss and chromosome 22q loss in approximately one-third of the cases (Suppl. Figure 3, online resource).

### Clear cell meningiomas are characterized by mutations in *SMARCE1*

**Fig. 2:**
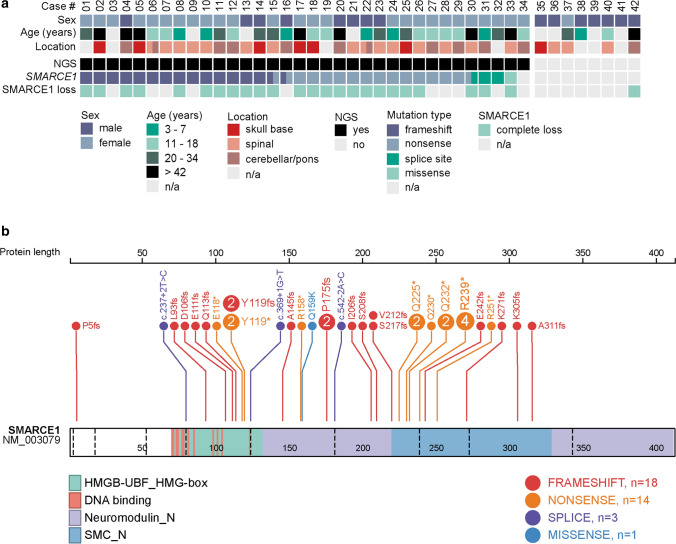
Clinicopathological characteristics and recurrent genetic alterations of the 42 clear cell meningiomas (**a**). Visualization of the *SMARCE1* mutation profile in the investigated cohort was created using the online tool ProteinPaint available at https://proteinpaint.stjude.org/ (**b**)

We next used targeted next-generation sequencing to gain insight into the mutational landscape of the meningiomas in this cluster and identified mutations in *SMARCE1* in 33 of the 34 cases (97%) with sufficient material available (Fig. 2 and Suppl. Table 1, online resource). Of the all in all 36 detected *SMARCE1* mutations (three cases harbored two separate mutations), 32 were nonsense or frameshift mutations predicted to result in complete loss of the protein product (Fig. 2 and Suppl. Table 1, online resource). Immunohistochemical detection of SMARCE1 showed widespread nuclear loss of expression in the tumor cells in all analyzed cases (*n* = 25). The mutant allele frequency for the *SMARCE1* mutations was highly variable (mean 58%; range 30–90%), suggesting a possible biallelic loss of the gene at least in some cases. In four of the cases with matched normal DNA available, the *SMARCE1* mutation was confirmed in the germline. Beside *SMARCE1*, no additional pathogenic mutations were detected (particularly none in the genes implicated in meningioma biology, including *NF2*, *AKT1*, *KLF4*, *TRAF7*, *SMO*, *SUFU*, *PTCH1*, *BAP1*, *SMARCB1*, *PTEN*, *PIK3CA* or *TERT* promoter mutations). In addition, screening of 705 meningioma samples with available sequencing and DNA methylation data revealed that *SMARCE1* mutations occurred exclusively within the specific epigenetic class of clear cell meningiomas.

### Differential gene expression segregates clear cell meningiomas and *NF2*-mutant meningiomas

**Fig. 3:**
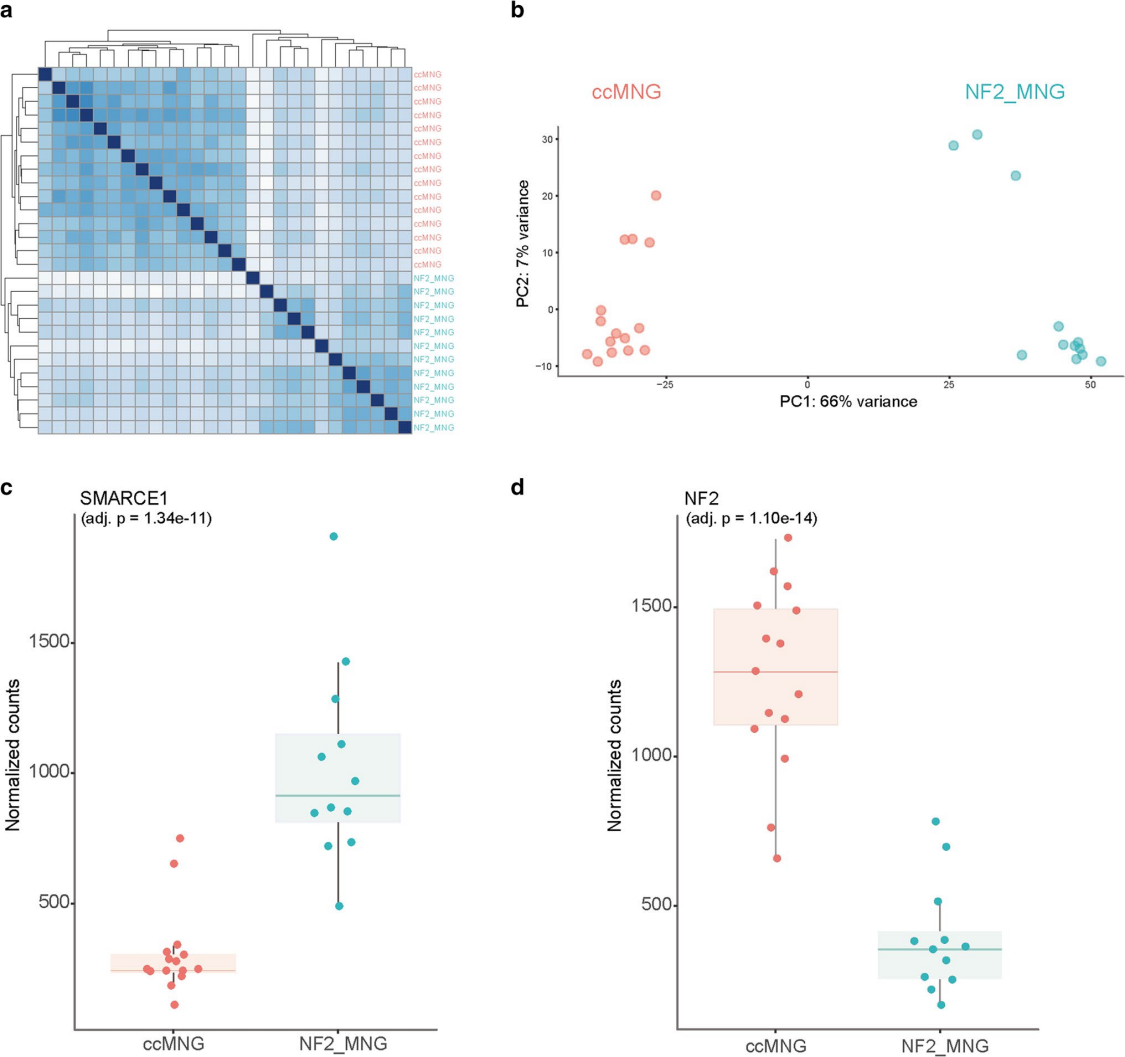
Differences in gene expression profiles between clear cell meningiomas and *NF2*-mutant meningiomas. Normalized transcript counts from clear cell meningioma and *NF2*-mutant meningioma samples clustered by Pearson’s correlation coefficient (**a**) and principal component analysis (**b**). *SMARCE1* (**c**) and *NF2* (**d**) expression in clear cell meningiomas (*n* = 15) determined by RNA-sequencing compared to *NF2*-mutant meningioma samples (*n* = 12)

We next performed mRNA sequencing of 15 tumor samples within the specific clear cell cluster and 12 *NF2*-mutant meningioma samples for comparison. Gene expression variability within the cohort did not correlate with clinical parameters or anatomical location. Unsupervised hierarchical clustering (Fig. 3a) and PCA analysis (Fig. 3b) demonstrated a clear segregation of tumor samples by DNA methylation profile and histology. In addition, quantification of mRNA expression confirmed decreased SMARCE1 expression in tumors within the clear cell cohort as compared to *NF2*-altered meningiomas (adjusted *p* = 1.34e-11; Fig. 3c). NF2 transcript levels were downregulated in *NF2*-mutant meningioma samples, respectively (adjusted *p* = 1.10e-14; Fig. 3d). Interestingly, analysis of EZH2, the catalytic subunit of the PRC2 complex which acts to antagonize the SWI/SNF complex, indicated an increased expression in clear cell meningiomas compared to meningiomas with *NF2* mutation (adjusted *p* = 1.70e-07; Suppl. Figure 4, online resource). Further differentially expressed genes between clear cell meningiomas and *NF2*-altered meningiomas are highlighted in supplementary Fig. 5 (online resource).

### Clinical characteristics and morphological features within the molecularly defined clear cell meningioma cohort

**Fig. 4:**
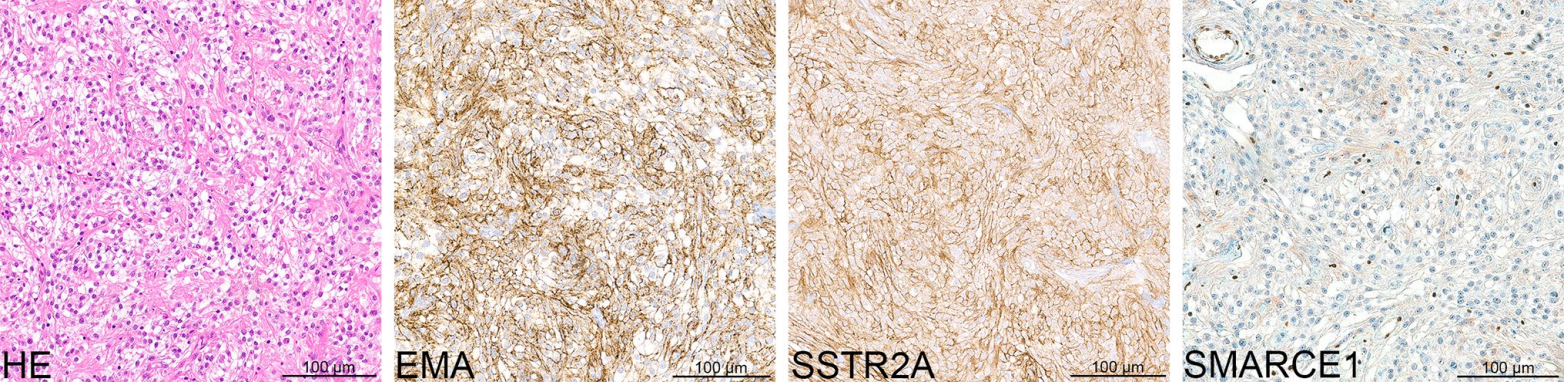
Morphological and immunohistochemical features of clear cell meningiomas within the cohort. H&E staining of one of the clear cell meningiomas included in the investigated cohort showing a high cellularity of round to polygonal clear cells with perivascular and interstitial collagen. Immunohistochemical expression of EMA and SSTR2A as well as nuclear loss of SMARCE1 in the tumor cells

**Fig. 5:**
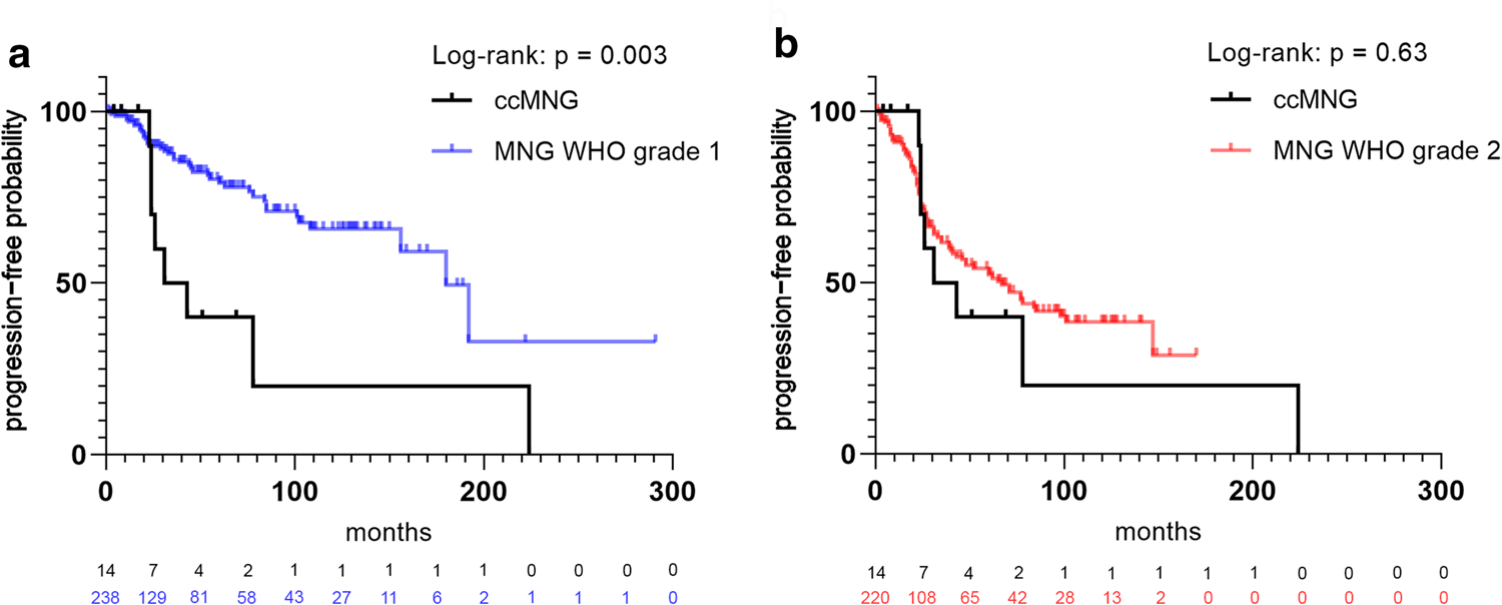
Time to progression or recurrence (TTP) of 14 patients from the investigated cohort (ccMNG) for whom follow-up data were available compared to TTP of 238 patients with meningioma WHO grade 1 (**a**) and 220 patients with meningioma WHO grade 2 (**b**)

The majority of the tumors in this molecular group (*n* = 29/42) were histologically diagnosed as clear cell meningioma (Suppl. Figure 1, online resource). Histopathological review according to the WHO 2016 classification of tumors of the central nervous system for all cases with sufficient material confirmed these findings. All these tumors histologically showed clearly recognizable cellular areas composed of round to polygonal clear cells with perivascular and interstitial collagen corresponding to the typical histological features of clear cell meningioma (Fig. 4). All tumors exhibited immunohistochemical expression of EMA and SSTR2A (Fig. 4 and Suppl. Figure 1, online resource). Mitotic counts were generally low to moderate (between 0 and 1.7 mitosis per mm^2^). Two tumors exhibited a higher count of up to 2.1 and 2.9 mitoses per mm^2^. The Ki-67 labeling index was very low (1–4%) in eight tumors, while 14 tumors had an elevated proliferation index of 5–10% and four of 15–20%. Consistent with previous reports [23], clear cell meningiomas in our series were located in the spine (*n* = 17) as well as intracranially (*n* = 18). Median age at presentation in our cohort was 25 years (range 3–75) with a female predominance (F:M ratio 1.8:1). Outcome data were available for 14 patients within the cohort. In comparison to a cohort of 458 meningiomas of other subtypes (WHO grade 1 and 2) the prediction of outcome seems to be consistent with the current WHO grade 2 assignment: Time to recurrence in patients with clear cell meningiomas in our cohort was significantly worse than in patients with WHO grade 1 meningiomas (*p* = 0.003; Fig. 5a) and not different from that in patients with other meningiomas of WHO grade 2 (*p* = 0.63; Fig. 5b).

## Discussion

Besides confirming the known association of *SMARCE1* mutations and clear cell meningioma, our findings indicate the existence of a highly distinct epigenetic signature of this meningioma subtype. Given previous studies in multiple tumor types on the robustness of cell-of-origin imprints in the epigenome of tumors [2, 6, 7, 25], this may suggest that these tumors arise from a different precursor cell population than the broad spectrum of the other meningioma subtypes. Interestingly, none of the other meningioma subtypes with characteristic alterations, e.g. mutations in *NF2*, *TRAF7/KLF4* or *BAP1*, or *YAP1* fusion, seem to form epigenetic clusters that are distinct to the same extent [19, 20]. However, given that *SMARCE1* is itself a broad epigenetic regulator, this could explain why the DNA methylation profile in *SMARCE1* deficient meningiomas is so different from that of other meningiomas. However, this finding will need confirmation in subsequent follow-up studies.

Although the clear cell subtype of meningioma represented the most common initial diagnosis in the primarily identified cohort, a smaller number of tumors were initially designated to other histological variants of meningioma (in particular diagnosed as atypical meningioma WHO grade 2). However, a histopathological review for all cases with sufficient material confirmed clearly recognizable cellular areas composed of round to polygonal clear cells with perivascular and interstitial collagen corresponding to the typical histological features of clear cell meningioma in each of the analyzed tumors. On the other hand, none of the histologically diagnosed clear cell meningiomas were found outside this specific cluster, which underlines a strong phenotype/genotype correlation. Therefore, in cases with diagnostic uncertainty immunostaining for SMARCE1 and DNA methylation profiling may be useful for accurate diagnosis of this subtype.

As *SMARCE1* mutations were detected in almost all tumors analyzed, it seems that clear cell meningiomas are primarily driven by these alterations. Germline loss-of-function mutations in *SMARCE1* were identified in familial spinal meningiomas in 2013 by Smith et al. [22] and seem to be highly linked to the clear cell subtype [23, 26]. *SMARCE1* encodes for a subunit of the SWI/SNF complex, which has been shown to play a widespread role in tumorigenesis [13, 15]. Thus, *SMARCE1* has been suggested as the potential oncogenic driver in clear cell meningioma [22]. Genetic alterations involving several other subunits of the SWI/SNF complex (in particular *SMARCB1* and *ARID1A*) are found in meningioma pathogenesis and seem to be associated with more aggressive subtypes of meningioma [3]. In addition, PRC2, which acts to antagonize the SWI/SNF complex, is upregulated in higher-grade meningiomas [3, 5], which is consistent with our results. Inactivating mutations in SWI/SNF subunits have been found in various other cancers, some of these demonstrating clear cell phenotype of the tumor cells as well [8, 17, 27, 28]. Interestingly, *SMARCE1* mutations seem to be restricted to this specific epigenetic class of clear cell meningiomas since no *SMARCE1* alterations were found in 705 other meningioma samples with available DNA methylation and sequencing data.

In line with our data, clear cell meningioma harboring a *SMARCE1* mutation have been commonly described in children and young adults [26]. Interestingly though, the age range in our cohort was relatively wide considering such a molecularly homogeneous tumor, suggesting a possible cell-of-origin, which is present throughout life. Analysis of time to progression or recurrence of these patients in comparison to those with meningioma WHO grade 2 revealed a similar outcome and hence supports the assignment of WHO grade 2 to these tumors. Whether germline mutations in the *SMARCE1* gene are associated with an increased risk for developing multiple meningiomas, as initially suggested [22], has to be determined in subsequent studies.

In summary, our data demonstrate a highly distinct epigenetic signature of clear cell meningiomas, that is associated with frequent mutations within the *SMARCE1* gene and/or loss of SMARCE1 protein expression, presumably by other mechanisms. Whether or not this has further implications for tumor classification as a subtype versus an entity requires further study.

## Supplementary Information

Below is the link to the electronic supplementary material.

Supplementary file1 (DOCX 13 KB)

Supplementary file2 (PDF 422 KB)
