# Supplementary material for: Clear cell meningiomas are defined by a highly distinct DNA methylation profile and mutations in SMARCE1
Source: Acta Neuropathol. 2020 Dec 14;141(2):281–90. doi: 10.1007/s00401-020-02247-2 (PMC7847462; doi:10.1007/s00401-020-02247-2)
Supplement: Supplementary file 1 — Supplementary file1 (DOCX 13 KB) [file 401_2020_2247_MOESM1_ESM.docx]

**Supplementary Information on Members of the German Consortium for Aggressive Meningiomas (KAM) beyond the authors**

**for**

**Clear cell meningiomas are defined by a highly distinct DNA methylation profile and mutations in *SMARCE1***

**Consortium Collaborators**

University Hospital Düsseldorf

Marc Remke, MD

Bastian Malzkorn, MD

Kerim Beseoglu, MD

University Hospital Hamburg

Franz Ricklefs, MD

Katrin Lamszus, MD

Manfred Westphal, MD

German Cancer Research Center Heidelberg

and

University of Augsburg

Matthias Schlesner, PhD

University Hospital Heidelberg

Christine Jungk, MD

Rolf Warta, PhD

Catharina Lotsch, MSc

University Hospital Magdeburg

Erol Sandalcioglu, MD

Elmar Kirches, PhD

University Hospital Munich

Jörg-Christian Tonn, MD

Christian Schichor, MD

Michael Schmutzer, MD

University Hospital Dresden

Gabriele Schackert, MD

Tareq Juratli, MD

Ortrud Uckermann, PhD

Matthias Kirsch, MD
