# Supplementary material for: Clear cell meningiomas are defined by a highly distinct DNA methylation profile and mutations in SMARCE1
Source: Acta Neuropathol. 2020 Dec 14;141(2):281–90. doi: 10.1007/s00401-020-02247-2 (PMC7847462; doi:10.1007/s00401-020-02247-2)
Supplement: Supplementary file 2 — Supplementary file2 (PDF 422 KB) [file 401_2020_2247_MOESM2_ESM.pdf]

Supplementary Table 1 - Summary of clinicopathological characteristics and key genetic alterations identified in the clear cell meningioma cohort

	Clinicla data				*Histopathology and immunohistochemistry*						*Molecular findings*	
**Case #**	**Sex**	**Age (years)**	**Location**	**Morphological subtype based on initial diagnosis**	**Revised morphological diagnosis**	**Grade 2 criteria according to WHO classification**	**EMA**	**SSTR2A**	**Ki-67**	**SMARCE1**	**NGS**	***SMARCE1***
1	f	20	n/a	chordoid meningioma	clear cell morphology (80%)	based solely on morphology	positive	positive	3%	loss	yes	SMARCE1:NM_003079:exon3:c.14delC:p.P5fs [90%]
2	f	53	skull base	clear cell meningioma	clear cell morphology (70%)	based solely on morphology	n/a	n/a	1%	loss	yes	SMARCE1:NM_003079:exon6:c.318delT:p.D106fs [68%]
3	f	n/a	n/a	meningioma not specified	n/a	n/a	n/a	n/a	n/a	n/a	yes	SMARCE1:NM_003079:exon7:c.525delT:p.P175fs [85%]
4	m	60	cerebellopontine angle	atypical meningioma	clear cell morphology (30%)	morphology, sheeting, prominent nucleoli, increased mitotic activity	positive	positive	20%	loss	yes	SMARCE1:NM_003079:exon9:c.726delA:p.E242fs [35%]
5	f	48	skull base	clear cell meningioma	clear cell morphology (70%)	based solely on morphology	positive	positive	15%	loss	yes	SMARCE1:NM_003079:exon6:c.278delT:p.L93fs [31%]
6	f	14	pontomedullary	clear cell meningioma	clear cell morphology (90%)	based solely on morphology	n/a	n/a	n/a	n/a	yes	SMARCE1:NM_003079:exon7:c.525delT:p.P175fs [90%]
7	f	17	spinal	clear cell meningioma	n/a	n/a	n/a	n/a	n/a	n/a	yes	SMARCE1:NM_003079:exon6:c.339delA:p.Q113fs [46%]
8	f	4	spinal	clear cell meningioma	clear cell morphology (60%)	based solely on morphology	positive	positive	7%	loss	yes	SMARCE1:NM_003079:exon7:c.432dup:p.A145fs [79%]
9	f	n/a	spinal	atypical meningioma	clear cell morphology (80%)	morphology, sheeting, increased cellularity, prominent nucleoli	n/a	n/a	n/a	n/a	yes	SMARCE1:NM_003079:exon8:c.624_627del:p.208_209del [88%]
10	f	4	spinal	clear cell meningioma	clear cell morphology (90%)	based solely on morphology	positive	positive	10%	loss	yes	SMARCE1:NM_003079:exon8:c.651_658del:p.217_220del [78%]
11	f	27	cerebellopontine angle	clear cell meningioma	clear cell morphology (90%)	based solely on morphology	positive	positive	7%	loss	yes	SMARCE1:NM_003079:exon9:c.811delA:p.K271fs [42%]
12	f	16	posterior fossa	clear cell meningioma	clear cell morphology (70%)	morphology, sheeting, increased cellularity, prominent nucleoli	positive	positive	10%	loss	yes	SMARCE1:NM_003079:exon10:c.933_945del:p.311_315del [47%]
13	m	n/a	spinal	clear cell meningioma	clear cell morphology (80%)	based solely on morphology	positive	positive	5%	loss	yes	SMARCE1:NM_003079:exon10:c.914dupA:p.K305fs [40%]
14	m	31	skull base	clear cell meningioma	clear cell morphology (80%)	based solely on morphology	positive	positive	5%	loss	yes	SMARCE1:NM_003079:exon8:c.634_659del:p.212_220del [30%]
15	f	21	spinal	chordoid meningioma	clear cell morphology (80%)	based solely on morphology	positive	positive	7%	loss	yes	SMARCE1:NM_003079:exon8:c.616delA:p.I206fs [36%] /// SMARCE1:NM_003079:exon9:c.C715T:p.R239X [39%]
16	m	3	posterior fossa	clear cell meningioma	n/a	n/a	n/a	n/a	n/a	n/a	yes	SMARCE1:NM_003079:exon6:c.331dupG:p.E111fs [45%] /// SMARCE1:NM_003079:exon6:c.C357A:p.Y119X [44%]
17	f	75	skull base	clear cell meningioma	clear cell morphology (80%)	based solely on morphology	positive	positive	4%	loss	yes	SMARCE1:NM_003079:exon8:c.C673T:p.Q225X [89%]
18	f	18	temporal	clear cell meningioma	clear cell morphology (95%)	based solely on morphology	positive	positive	10%	loss	yes	SMARCE1:NM_003079:exon7:c.C472T:p.R158X [55%]
19	f	13	intradural	clear cell meningioma	clear cell morphology (95%)	based solely on morphology	positive	positive	1%	loss	yes	SMARCE1:NM_003079:exon9:c.C715T:p.R239X [52%]
20	m	60	cerebellopontine angle	clear cell meningioma	clear cell morphology (90%)	based solely on morphology	positive	positive	3%	loss	yes	SMARCE1:NM_003079:exon9:c.C715T:p.R239X [37%]
21	m	n/a	spinal	clear cell meningioma	clear cell morphology (95%)	based solely on morphology	n/a	n/a	3%	loss	yes	SMARCE1:NM_003079:exon8:c.C688T:p.Q230X [58%]
22	m	3	spinal / medulla oblongata	clear cell meningioma	clear cell morphology (90%)	based solely on morphology	positive	positive	15%	loss	yes	SMARCE1:NM_003079:exon8:c.C694T:p.Q232X [90%]
23	m	34	spinal	angiomatous meningioma	clear cell morphology (30%), high proportion of blood vessels	based solely on morphology	positive	positive	2%	loss	yes	SMARCE1:NM_003079:exon8:c.C694T:p.Q232X [33%]
24	f	7	spinal	atypical meningioma	clear cell morphology (30%)	morphology, sheeting, prominent nucleoli, increased mitotic activity	n/a	n/a	15%	loss	yes	SMARCE1:NM_003079:exon9:c.C715T:p.R239X [89%]
25	f	23	skull base	clear cell meningioma	clear cell morphology (90%)	based solely on morphology	positive	positive	5%	loss	yes	SMARCE1:NM_003079:exon6:c.C357G:p.Y119X [67%]
26	f	12	spinal	clear cell meningioma	clear cell morphology (70%)	based solely on morphology	positive	positive	7%	loss	yes	SMARCE1:NM_003079:exon6:c.G352T:p.E118X [33%]
27	f	16	cerebellopontine angle	meningioma not specified	clear cell morphology (70%)	based solely on morphology	n/a	n/a	n/a	n/a	yes	SMARCE1:NM_003079:exon6:c.356dupA:p.Y119_E120delinsX [37%]
28	f	17	spinal	clear cell meningioma	n/a	n/a	n/a	n/a	n/a	n/a	yes	SMARCE1:NM_003079:exon8:c.C673T:p.Q225X [82%]
29	f	11	cerebellum	clear cell meningioma	clear cell morphology (80%)	based solely on morphology	n/a	n/a	n/a	n/a	yes	SMARCE1:NM_003079:exon9:c.C751T:p.R251X [86%]
30	f	51	spinal	clear cell meningioma	clear cell morphology (70%)	based solely on morphology	positive	positive	10%	loss	yes	SMARCE1:NM_003079:exon6:c.357delC:p.Y119X [33%] /// SMARCE1:NM_003079:splicing:c.542-2A>C [36%]
31	f	7	posterior fossa	clear cell meningioma	clear cell morphology (70%)	based solely on morphology	positive	positive	1%	loss	yes	SMARCE1:NM_003079:splicing:c.369+1G>T [66%]
32	f	20	n/a	clear cell meningioma	n/a	n/a	n/a	n/a	n/a	n/a	yes	SMARCE1:NM_003079:splicing:c.237+2T>C [51%]
33	f	71	spinal	clear cell meningioma	clear cell morphology (90%)	based solely on morphology	positive	positive	10%	loss	yes	SMARCE1:NM_003079:exon7:c.C475A:p.Q159K [48%]
34	f	12	cerebellopontine angle	clear cell meningioma	clear cell morphology (70%)	based solely on morphology	n/a	n/a	n/a	n/a	yes	-
35	m	n/a	skull base	meningioma not specified	n/a	n/a	n/a	n/a	n/a	n/a	no	n/a
36	m	n/a	spinal	atypical meningioma	n/a	n/a	n/a	n/a	n/a	n/a	no	n/a
37	f	26	spinal	clear cell meningioma	clear cell morphology (70%)	based solely on morphology	n/a	n/a	5%	n/a	no	n/a
38	m	6	n/a	n/a	n/a	n/a	n/a	n/a	n/a	n/a	no	n/a
39	m	n/a	n/a	n/a	n/a	n/a	n/a	n/a	n/a	n/a	no	n/a
40	m	16	spinal	clear cell meningioma	n/a	n/a	n/a	n/a	n/a	n/a	no	n/a
41	m	n/a	n/a	n/a	n/a	n/a	n/a	n/a	n/a	n/a	no	n/a
42	m	42	cerebellopontine angle	clear cell meningioma	clear cell morphology (95%)	based solely on morphology	positive	positive	10%	loss	no	n/a

Abbreviations: m, male; f, female; n/a, data not available; NGS, next generation sequencing; [ ], mutant allele fraction in percentage

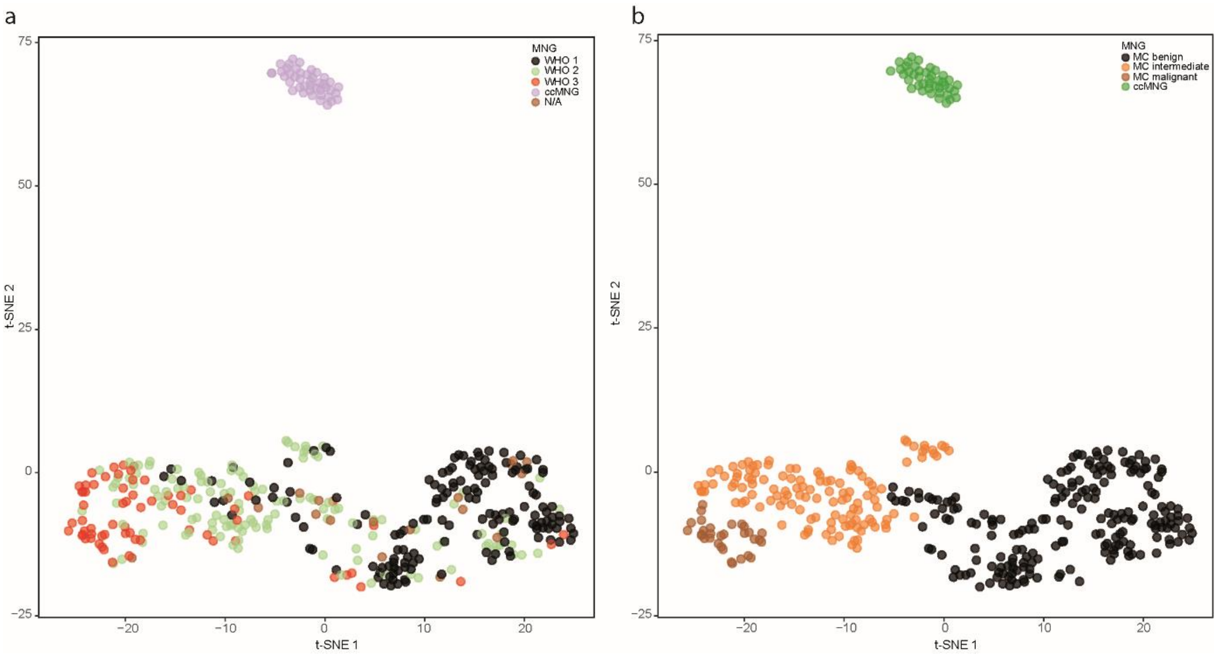

Supplementary Fig. 1 t-distributed stochastic neighbor embedding (t-SNE) analysis of DNA methylation profiles of the 42 clear cell meningiomas (ccMNG) alongside selected reference meningioma samples of different WHO grades (a) and DNA methylation classes (b).

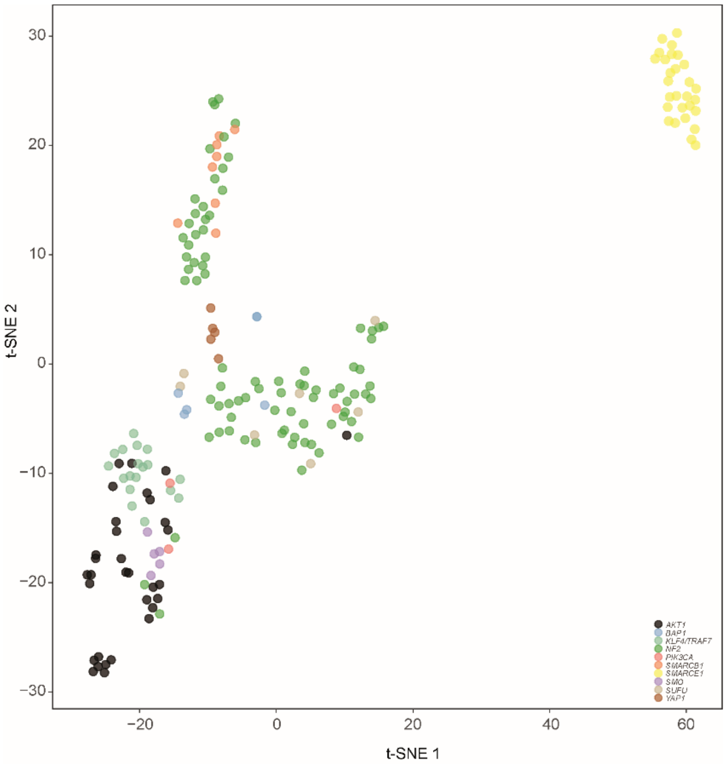

Supplementary Fig. 2 t-distributed stochastic neighbor embedding (t-SNE) analysis of DNA methylation profiles of meningioma samples with different alterations.

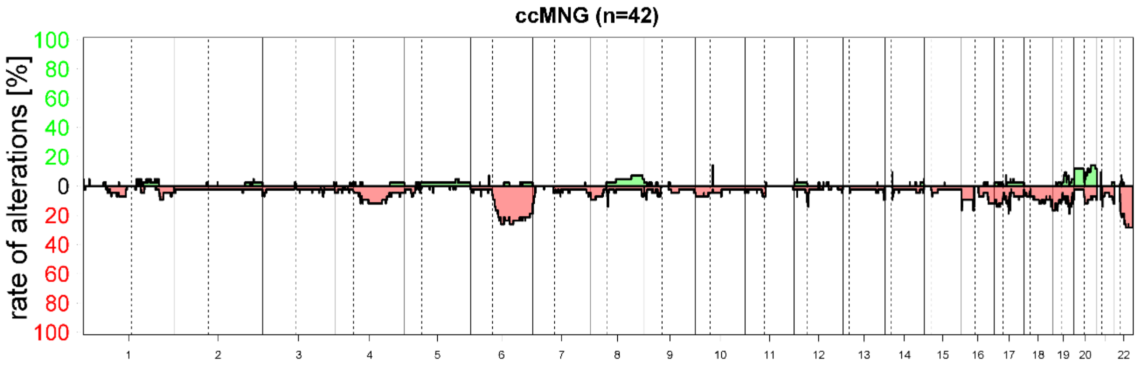

Supplementary Fig. 3 Integrated copy number plots derived from DNA methylation array data for the 42 clear cell meningiomas (ccMNG).

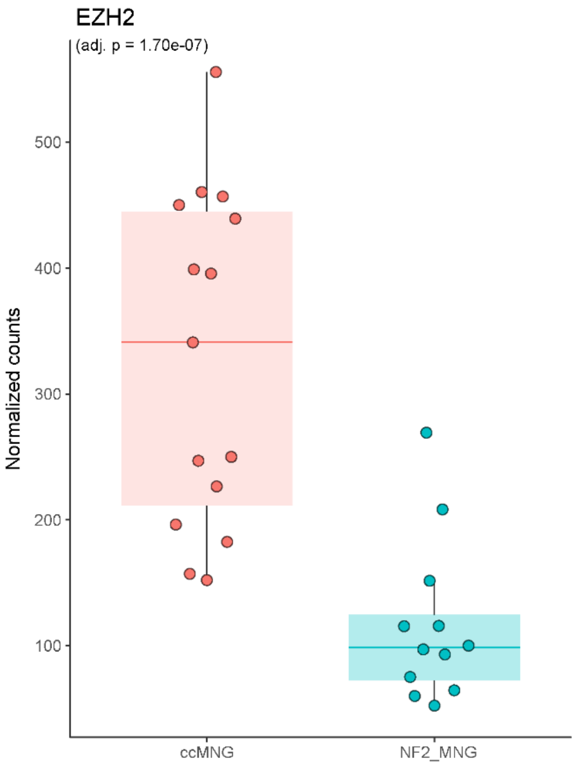

Supplementary Fig. 4 *EZH2* expression in clear cell meningiomas (n=15) determined by RNA-sequencing compared to *NF2*-mutant meningioma samples (n=12).

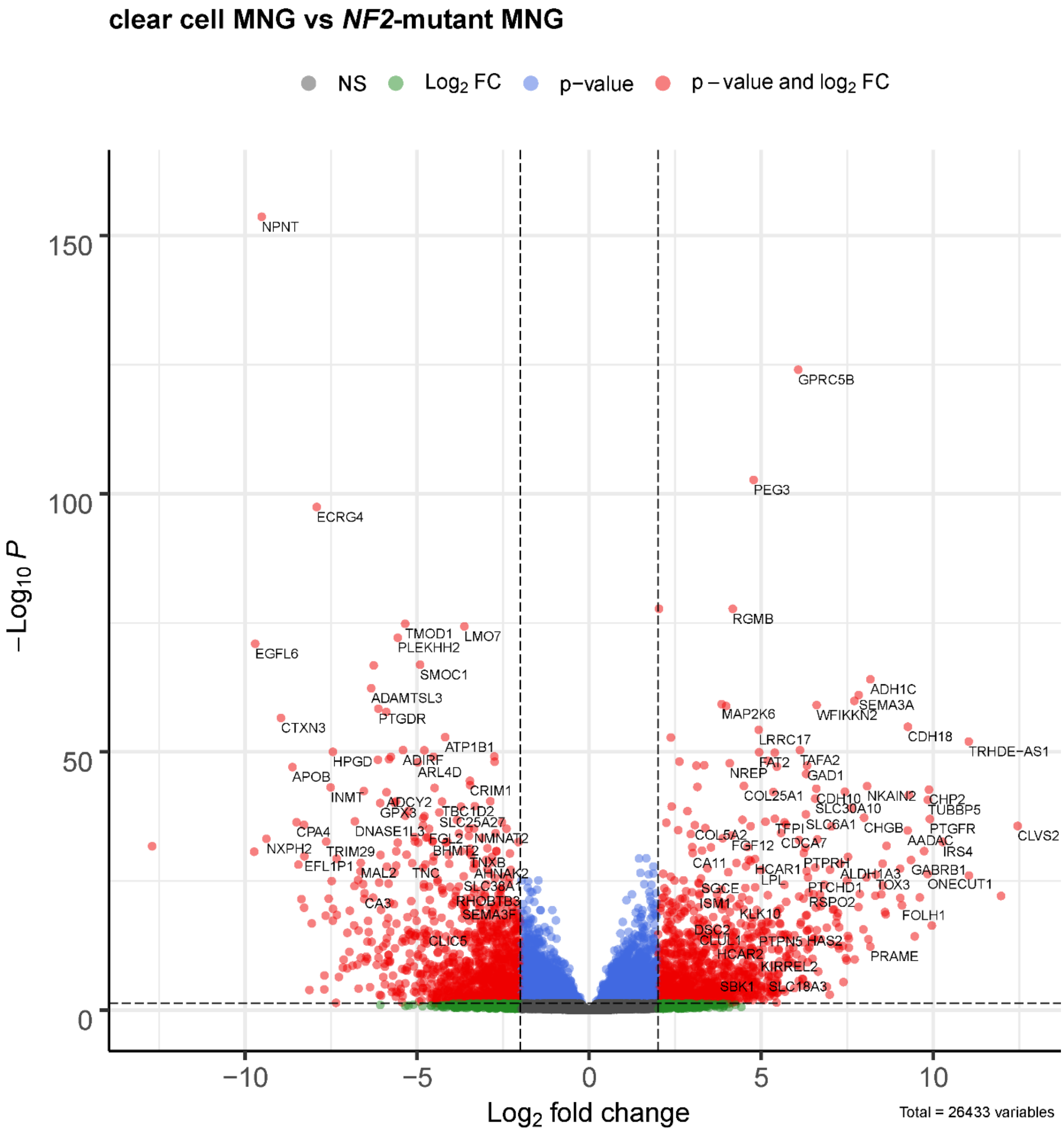

Supplementary Fig. 5 Volcano plot illustrating differences in gene expression between clear cell meningiomas and *NF2*-mutant meningiomas.
